# Fabrication of Metallic Biomedical Scaffolds with the Space Holder Method: A Review

**DOI:** 10.3390/ma7053588

**Published:** 2014-05-06

**Authors:** Budi Arifvianto, Jie Zhou

**Affiliations:** Biomaterials Technology Section, Department of Biomechanical Engineering, Faculty of Mechanical, Maritime and Materials Engineering, Delft University of Technology, Mekelweg 2, 2628 CD, Delft, The Netherlands

**Keywords:** tissue engineering, scaffold, space holder method, powder metallurgy, titanium

## Abstract

Bone tissue engineering has been increasingly studied as an alternative approach to bone defect reconstruction. In this approach, new bone cells are stimulated to grow and heal the defect with the aid of a scaffold that serves as a medium for bone cell formation and growth. Scaffolds made of metallic materials have preferably been chosen for bone tissue engineering applications where load-bearing capacities are required, considering the superior mechanical properties possessed by this type of materials to those of polymeric and ceramic materials. The space holder method has been recognized as one of the viable methods for the fabrication of metallic biomedical scaffolds. In this method, temporary powder particles, namely space holder, are devised as a pore former for scaffolds. In general, the whole scaffold fabrication process with the space holder method can be divided into four main steps: (i) mixing of metal matrix powder and space-holding particles; (ii) compaction of granular materials; (iii) removal of space-holding particles; (iv) sintering of porous scaffold preform. In this review, detailed procedures in each of these steps are presented. Technical challenges encountered during scaffold fabrication with this specific method are addressed. In conclusion, strategies are yet to be developed to address problematic issues raised, such as powder segregation, pore inhomogeneity, distortion of pore sizes and shape, uncontrolled shrinkage and contamination.

## 1. Introduction

In recent years, bone tissue engineering as an alternative approach to bone defect reconstruction has received increasing attention within the biomedical research community. With this approach, damaged bone tissue can be repaired and remodeled with new bone cells in a scaffold implanted at the defect site [1]. This new approach is considered highly promising in overcoming the major deficiencies of autogeneous bone grafts that have been used for many years as gold standard implants to support the formation of new bone cells [2]. Autogeneous bone grafts contain living cells that can differentiate to osteoblasts for bone tissue regeneration (osteogenic). They also encourage local or additional cells to differentiate to osteoblasts (osteoinductive) and serve as templates that support newly formed bone cells (osteoconductive) [2]. Their clinical applications can however be seriously limited by disease transmission and infection from donor to recipient, donor site morbidity, bone defect size, viability of the host bed and availability [2,3]. These limitations have stimulated the development of synthetic materials for scaffolds that may eventually replace autogeneous grafts [4–6].

Scaffolds for bone tissue engineering are usually designed with porous structures to facilitate cellular activities, such as the migration and proliferation of osteoblasts and mesenchimal cells as well as the transport of nutrients and oxygen required for vascularization during bone tissue development [4,5,7]. With bone tissue grown and developed appropriately in porous structures, osseointegration and stability of implants are improved, as indicated by increased fixation strength as a result of mechanical interlocking between porous implants and surrounding host bone tissue [5,8].

Bone tissue engineering scaffolds have been made from various materials [6]. Ceramic biomaterials such as hydroxyapatite and β-tricalcium phosphate [9] are well known for their biocompatibility and bioactivity, but they are too brittle for applications where sufficient mechanical strength and fracture toughness are required [5,6,10]. Polymeric biomaterials are biocompatible and biodegradable, but most of them possess poor mechanical properties [6]. Metallic scaffolds have been acknowledged as the most suitable materials for porous implants for bone tissue engineering. Titanium, magnesium and their alloys, for example, have demonstrated a combination of excellent mechanical properties and biocompatibility [11–14], allowing their use as load-bearing implants and bone tissue scaffolds [15–18]. Biodegradability of metallic biomaterials such as magnesium and its alloys has, in recent years, received immense attention [14] and their clinical applications will become widespread when degradation rate can be controlled and mechanical integrity can be assured over a period of time as clinically required. The major limitation of other metallic biomaterials such as stainless steel and cobalt-chromium alloys is related to their high elastic moduli that lead to stress shielding and bone resorption over time. With porous structure, however, the mismatch in elastic modulus between metallic implant and host bone tissue can be reduced [6,19].

Currently, various methods for the fabrication of metallic scaffolds have been presented in the literature, for example, powder sintering, expansion of pressurized gas bubbles, powder deposition, rapid prototyping and space holder method [6,19–21]. Powder sintering, as a traditional powder metallurgy method, has been widely used for its simplicity in generating porous structures needed for scaffolds. With this method, metal powder particles are compacted and sintered. Pores are formed from the interstices of powder particle arrangements [20,22–24]. As such, pore sizes and pore shape of sintered powder compacts depend on the sizes and shape of starting powder particles [20,22,23] and maximum porosity achievable is limited to 35% [23]. Powder sintering may be coupled with the space holder method so as to reach higher porosity levels and better control over porous structure in scaffolds [25,26].

**Figure 1.**
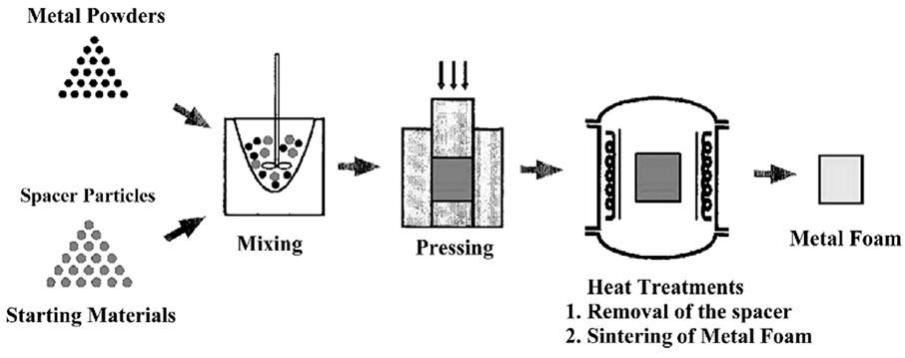
Schematic illustration of fabrication route of metallic scaffold with the space holder method. Reprinted with permission from [25]. Copyright 2001, Elsevier.

Scaffold fabrication with the space holder method relies on temporary particles added to metallic matrix powder, *i.e.*, space holding particles that act as a pore former. Figure 1 schematically illustrates the main steps involved in metallic scaffold fabrication with the space holder method. Space-holding particles are first mixed and compacted together with metallic matrix powder particles and then removed either before or during sintering, leaving new pores behind in the matrix. The space holder method may also be called the fugitive filler method [27], considering the similarities in the principle of these two fabrication methods. A number of space holder materials have been utilized, such as carbamide (CO(NH_2_)_2_) [28–32], ammonium hydrogen carbonate (NH_4_HCO_3_) [33,34], sodium chloride (NaCl) [35,36], starch [37,38], saccharose [39], polymethyl-methacrylate (PMMA) [40], magnesium (Mg) [41,42], steel [43] and paraformaldehyde [44].

**Figure 2.**
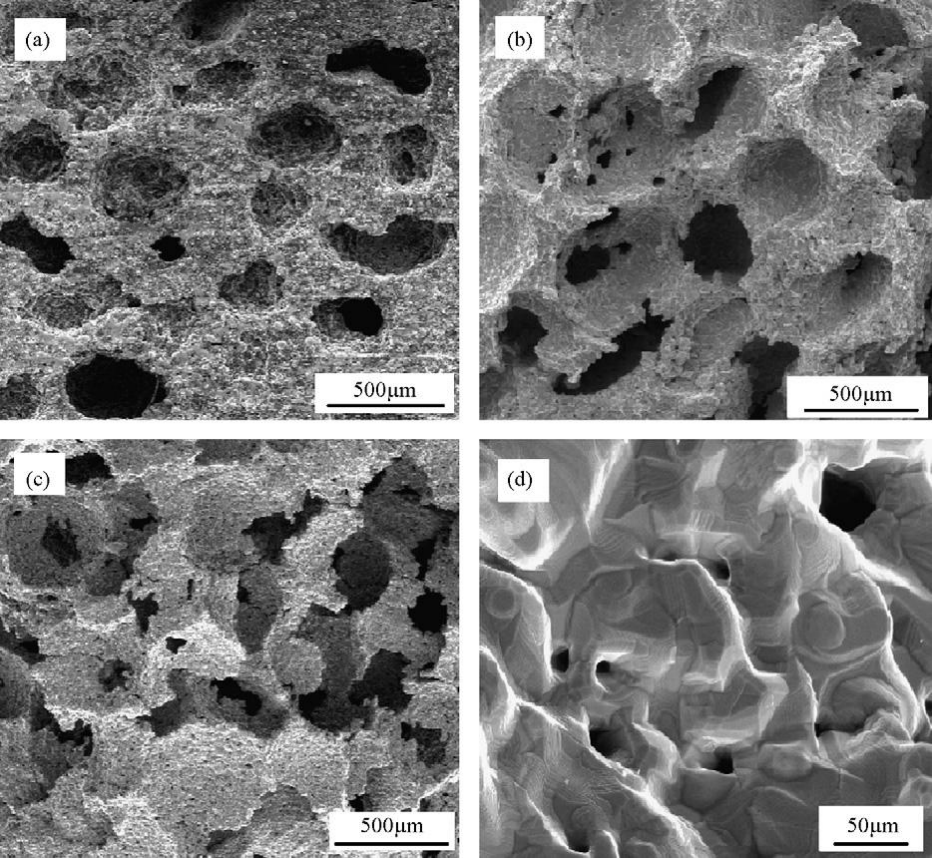
Macro-pores of titanium scaffold with porosity of (**a**) 55%; (**b**) 70% and (**c**) 75% and (d) micro-pores in the scaffold cell walls. Reprinted with permission from [28]. Copyright 2009, Elsevier.

**Figure 3.**
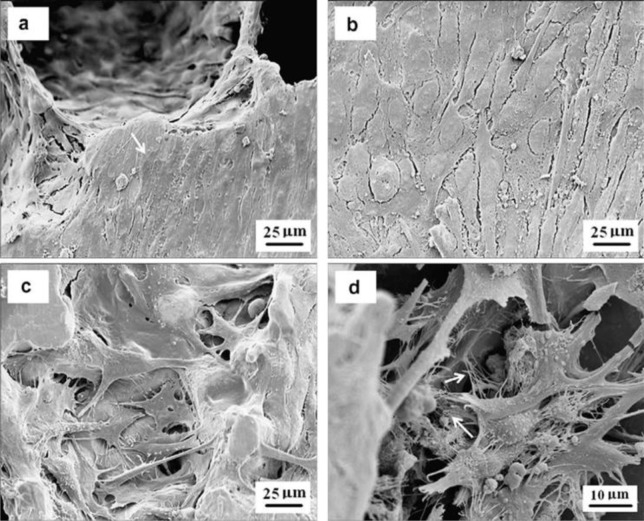
Osteoblast cells after 14 days culture formed in Ti-Nb-Zr alloy scaffold prepared with the space holder method: (**a**) cell formed in pores and surface of the scaffold; (**b**) a cell layer on the surface of scaffold; (**c**) cell formed in the pores of scaffold and (**d**) cells that were formed in the space between particles. Reprinted with permission from [45]. Copyright 2009, Elsevier.

Earlier designed metallic scaffolds produced with the space holder method exhibited characteristics and performance that could meet the criteria of scaffolds for bone tissue engineering, *i.e.*, high porosity (45%–80%), interconnected pores, appropriate pore sizes (200–500 μm) and adequate mechanical properties in terms of elastic modulus and compressive strength [25]. Figure 2 shows the porous structures of titanium scaffolds having porosities of 55%–75% processed by using the space holder method [28]. A series of *in vitro* cell compatibility tests, such as those shown in Figure 3, showing bone cell attachment, proliferation and differentiation in the Ti-Nb-Zr alloy scaffold, confirmed the biocompatibility of the scaffold produced with the space holder method [45]. This finding was attributed to the ability of the space holder method to produce high-porosity scaffolds (up to 70%) with interconnected pores, considering the great importance of porosity for bone cell activities [32,46].

Up till now, many studies have been conducted on scaffold fabrication with the space holder method and on the determination of scaffold performance. Although a large number of papers on the subject have been published, the procedures used for scaffold fabrication have not yet been collated. Moreover, many of the papers on the performance of metallic scaffolds as well as on the fabrication procedures overlap each other, resulting in difficulties in tracking the research progress and in establishing standardized procedures for metallic scaffold fabrication with this method.

Recently, several review articles on scaffold materials and fabrication technology were published and these articles all mention the space holder method as one of the effective methods for the fabrication of metallic scaffolds [6,19,20,47,48]. Dunand [20] and Singh *et al.* [19], for example, presented various techniques for the fabrication of titanium foams for structural and biomedical applications. Ryan *et al.* [6] compared similar fabrication techniques for various metallic scaffolds and the characteristics of resulting scaffolds as well as their influences on clinical performance. The fabrication of porous NiTi scaffolds from powders was reviewed by Bansiddhi and Dunand [47]. By extracting information from these articles, it becomes clear that scaffold fabrication with the space holder method involves the following major steps: (i) mixing of metal matrix powder and space-holding particles; (ii) compaction of granular materials obtained from mixing; (iii) removal of space-holding particles from compacted granular materials and (iv) sintering of scaffold preform [6,19,20]. Despite a clear description on the general principle of the method, these review articles do not provide detailed information on the procedures at these steps or the hurdles that remain concerning large-scale fabrication of scaffolds with controllable, reproducible mechanical properties and architectural characteristics.

In this paper, detailed information on the procedures for the fabrication of metallic biomedical scaffolds with the space holder method is presented. With a specific focus on this method, the review paper is intended to supplement the previous review papers on the fabrication of metallic scaffolds or foams with the powder metallurgy approach [6,19–21]. On the basis of a critical review of a large number of papers published since 2000, technical capabilities of this method, its limitations and challenges are elucidated. Considering the fact that the subject of this review paper is an interdisciplinary one, relevant research articles on general powder technology and biomaterials are referred to so that readers can access the fundamentals quickly and as required.

## 2. Powder Selection and Preparation

The fabrication for metallic scaffolds with the space holder method begins with the mixing of metal matrix powder and space-holding particles (see Figure 1). It is important to note that appropriate matrix powder particles must be selected and prepared, before scaffold fabrication can be started. This is mainly because the properties of the resultant scaffolds are to some extent dictated by the characteristics of both metal matrix powder and space-holding particles [22,34,49–52].

### 2.1. Elemental and Alloyed Matrix Powders

Basically, the porous structure of a scaffold stems from the arrangements of metal matrix powder particles that build up the scaffold framework. Pure titanium and magnesium are among powdered metallic materials that have been processed as the matrices of scaffolds [25,26,53]. Since the mechanical and biological properties of these pure metallic scaffolds are often not satisfactory, scaffolds prepared from alloy powders, such as Ti-6Al-4V [22,42,54], NiTi [36,55,56], Ti-6Ta-4Sn [57], Ti-5Mn [58], Ti-7.5Mo [59,60], Ti-10Mo [61], AZ91 [62] and Mg-Zn [63] are preferred.

In general, there are two techniques that may be used for the preparation of alloy powders, *i.e.*, the pre-alloying (PA) technique and blended elemental (BE) technique [64]. In the PA technique, the addition of one or more elements to the base metal is performed before the alloy is atomized into a powder form. By contrast, the BE technique is deployed by blending elemental powder particles in, e.g., a planetary ball milling machine running at a prescribed ball-to-powder ratio for a certain duration. During the blending process, elemental powder particles are plastically deformed and cold welded [59,60] and alloying takes place during sintering after cold compaction of blended elemental powders, in addition to alloying that may have already taken place during intensive mixing.

Alloying elements must be carefully selected, based on the considerations on possible toxic and allergic side-effects [13]. Several alloying elements have been found to be toxic and inappropriate for biomedical applications, such as aluminum (Al), nickel (Ni), iron (Fe), vanadium (V) and cobalt (Co) [45], while other elements such as zirconium (Zr), niobium (Nb) and tantalum (Ta), molybdenum (Mo) and tin (Sn) have been taken as safe alloying elements [13,45]. Table 1 shows a number of examples of scaffolds that have been developed from powdered alloys with the space holder method.

### 2.2. Morphology of Matrix Powder Particles

**Figure 4.**
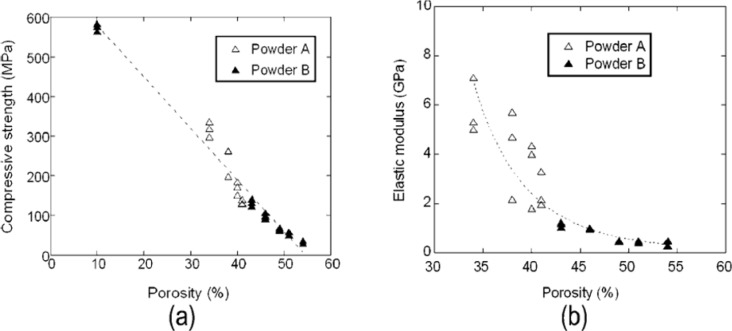
(**a**) Compressive strength and (**b**) elastic modulus of sintered Ti-6Al-4V scaffolds prepared with spherical (Powder A) and angular (Powder B) powder particles. Reprinted with permission from [22]. Copyright 2008, Wiley Periodicals.

The properties of a metallic scaffold are influenced by the morphological characteristics of metal matrix powder particles. Guden *et al.* [22] reported higher porosity and larger pore sizes, as a result of the sintering of a compacted angular powder, in comparison with a compacted spherical powder. The compressive strength and elastic modulus of the sintered angular matrix powder were lower than those of the sintered spherical powder [22]. Figure 4 shows the relationships between porosity and mechanical properties, *i.e.*, (a) compressive strength and (b) elastic modulus of scaffolds prepared from the sintered spherical (A) and angular (B) Ti-6Al-4V powders. With increasing porosity, the mechanical properties of the scaffolds deteriorated. The better mechanical properties of the scaffolds from the sintered spherical powder were attributed to lower porosity, as a result of a higher deformation capacity of spherical particles than that of angular ones under the same compacting pressure [22]. It is important to distinguish the mechanical properties in the sintered state from those in the green state (after compaction). Interestingly, the use of angular matrix powder particles led to a higher green strength of scaffolds [49], while the green body of scaffolds prepared from compacted spherical powder particles tended to collapse, especially during the removal of space-holding particles, due to less mechanical interlocking between initially spherical powder particles in compacts.

### 2.3. Sizes of Matrix Powder Particles

The quality of a sintered scaffold and densification during sintering depend on the sizes of matrix powder particles. Bram *et al.* [50] showed fully densified scaffold framework as a result of the sintering of powder particles having sizes finer than 16 μm. However, the sintering of powder particles of larger sizes resulted in scaffold framework with voids and sintering necks [50]. In addition, Chen *et al.* [51] showed downsizing titanium particles through ball milling from 19.79 to 5.89 μm significantly increased the surface energy and apatite-inducing ability of the resulting titanium scaffolds.

### 2.4. Types of Space-Holding Particles

As the porous architecture and mechanical properties of scaffolds are greatly affected by space-holding particles used in scaffold fabrication, the properties and geometrical characteristics of space-holding particles must be considered. The selection of space-holding particles must be based on the following criteria:

- biocompatibility and non-cytotoxicity
- chemical stability
- removal capability
- mechanical properties.

In order to minimize the adverse effects on the resultant scaffolds due to contamination by the residues of space-holding particles, Bor and his co-workers and Kim *et al.* [41,42,67–72] used a pure magnesium powder as the space holder. In addition, food-grade powders, such as sodium chloride [35,36,55,73,74], saccharose [39], dextrin corn starch [38] and tapioca starch [37] have been devised as safe space holders. Reactions between matrix powder and space-holding particles or binder must be avoided and hence chemically stable space-holding particles are preferred. Any reaction between decomposed space-holding particles and scaffold framework may deteriorate the mechanical properties of the resulting scaffolds [72]. Moreover, reactions between space-holding particles and binder that is used in the fabrication process must also be avoided, because they may distort the shape and sizes of space-holding particles and the resulting macro-pore geometry of scaffolds. In addition, space-holding particles must be able to be removed quickly from scaffold preforms in order to prevent contaminations by space holder residues. Finally, the strength of space holder material is critical, because it is related to the possibility of deformation and then breakage that may occur during the compaction process (see Section 4). Considering these criteria, metallic powders such as magnesium and steel whose mechanical properties are better than organic space holders, such as carbamide and sodium chloride, have been used [41,43]. Table 2 shows a few examples of space holders and the considerations in selecting these materials for metallic biomedical scaffolds.

### 2.5. Sizes of Space-Holding Particles

**Figure 5.**
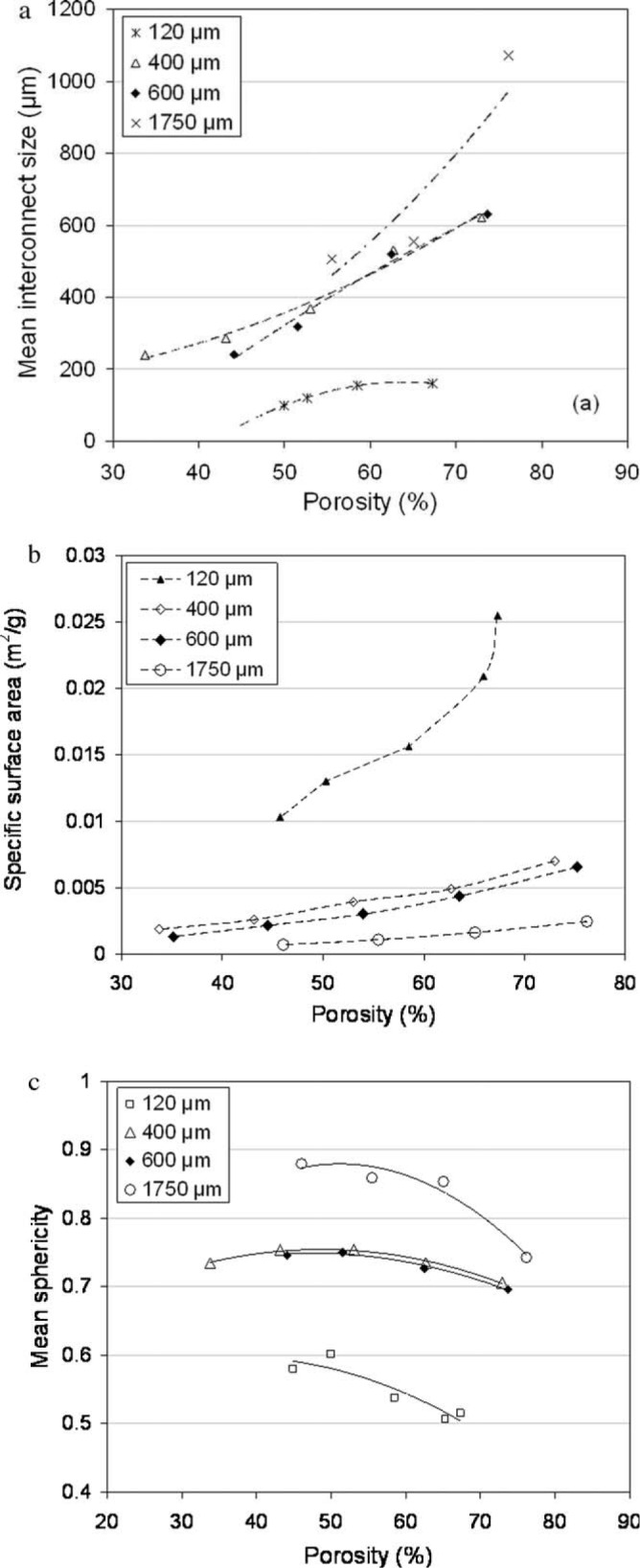
(**a**) Mean interconnect sizes; (**b**) surface area and (**c**) mean pore sphericity as a function of the porosity of scaffolds prepared using space holders of different particle sizes. Reprinted with permission from [49]. Copyright 2011, Elsevier.

The sizes of space-holding particles must be selected, based on the desired macro-pore sizes in scaffolds. Spacer powder particles having sizes in a range of 100–500 μm are commonly chosen to produce scaffolds with macro-pore sizes of 300–400 μm. In addition, appropriate sizes of space-holding particles can result in the formation of interconnected macro-pores in scaffolds. By means of tomographic analysis, Tuncer *et al.* [49] successfully studied the influence of space-holding particle size on the 3-dimensional porous architecture of titanium scaffolds (see Figure 5). As shown in Figure 5a, macro-pore interconnect size in scaffolds increases, when space-holding particles of a larger size are used. This could be attributed to greater packing coordination of larger space-holding particles, as compared with smaller particles after compaction.

Greater porosity is then produced in scaffolds. As a consequence of having a larger macro-pore interconnect size, the specific surface area of scaffolds decreases, when space-holding particles of a larger size are used (Figure 5b). Moreover, macro-pore sphericity increases when space-holding particles of a larger size are used (Figure 5c). Pore wall thickness increases with increasing space-holding particle size for the same relative density, compared to the one with space-holding particles having a smaller size, resulting in better mechanical properties of scaffolds.

### 2.6. Morphology of Space-Holding Particles

Regarding the shape of space-holding particles, Bekoz and Oktay [52] showed decreased compressive strength and elastic modulus of stainless steel foams with the use of an irregularly shaped carbamide space holder. Stainless steel foams with spherical pores exhibited better mechanical properties, as they had smoother pore surfaces that could minimize stress concentrations due to a decreased number of sharp edges in scaffolds. Zhang *et al.* [77] confirmed that spherical porogens (space holder) resulted in polymeric scaffolds with higher compressive strength and elastic modulus, corresponding to a smaller number of defects in scaffolds, compared to those processed with cubical porogens. At high porosity levels, the spherical space holder yielded a more ordered array of macro-pores and interconnections in the interior of scaffolds. With the cubical space holder, however, effective geometrical packing in scaffold preforms could not be achieved, resulting in an irregular porous structure.

### 2.7. Size Distribution of Space-Holding Particles

The size distribution of space-holding particles must be controlled. In most cases, a narrow distribution of space-holding particle sizes is preferred. In order to investigate the effect of the particle size distribution of the space holder, Li *et al.* [34] compared porous architecture and mechanical properties of NiTi scaffolds prepared with sieved and non-sieved space-holding particles. Through sieving, the size distribution of space-holding particles could be narrowed down to a certain range. From this study, it was found that NiTi scaffolds processed with the non-sieved space holder exhibited lower strength and possessed nearly no superplastic properties, resulting from randomly distributed macro-pores in scaffolds. This finding confirms the importance of the size uniformity of space holding particles to the performance of metallic scaffolds.

## 3. Mixing

With the space holder method, mixing of matrix powder and space-holder is conducted as the first step in scaffold fabrication. Porosity and pore interconnectivity of resultant scaffolds can be determined by adjusting the volume fraction of space-holding particles. The relationship between porosity *P* and the volume fraction of space-holding particles is expressed mathematically in Equation (1),

$$P=\frac{V_{\text{pore}}}{V_{\text{sc}}}\approx \frac{V_{\text{sh}}}{V_{\text{scp}}}$$ (1)

$$P=\frac{\left(m_{\text{sh}}/\rho_{\text{sh}}\right)}{\left(m_{\text{sh}}/\rho_{\text{sh}}+m_{\text{m}}/\rho_{\text{m}}\right)}$$ (2)

where *V*_pore_ and *V*_sc_ are the pore volume and total volume of the scaffold, respectively; whereas *V*_sh_ and *V*_scp_ are the volume space-holding particles and total volume of the scaffold preform, respectively. Using Equation 2, scaffold porosity can be defined from the mass *m* and density ρ of metal matrix powder and space-holding particles. The subscripts sh and m in Equation (2) correspond to space holder and matrix powder, respectively. Porosity levels of scaffold products are however often found to deviate from designed values, mainly due to micro-pores in scaffold framework and low mixing efficiency. Detailed information on micro-pores is presented in Section 6. Low mixing efficiency can be attributed to powder agglomeration and adhesion of mixed powder particles to the inner surface of the mixing container wall. As a consequence, the resulting mixture is less uniform, consisting of granular materials with inappropriate homogeneity.

### 3.1. Effect of Mixing on Porosity and Pore Distribution

With the space holder method, both open and closed pores can be formed in scaffolds, depending on the volume fraction of space-holding particles added to the mixture [44]. Open pores are built up from coalesced space-holding particles as a consequence of the compaction process, while closed pores are formed from isolated space-holding particles in the mixture. The number of such isolated pores increases as space holder content decreases [53]. Bhattarai *et al.* [46] observed open pores with interconnected channels in titanium scaffolds at a porosity level of 70%. Poor pore interconnection was achieved in other scaffolds at lower porosity levels [46]. Sharma *et al.* [78] revealed that the transition from closed or isolated pores to interconnected pores occurred when the total porosity of scaffolds reached 55%.

The importance of the mixing process on the pore distribution of scaffolds was recently emphasized [34,78,79]. As macro-pores are formed from the space occupied by space-holding particles, homogeneous distribution of these particles in the mixture will lead to homogeneous macro-pore distribution in scaffolds. Furthermore, as discussed earlier, the mechanical properties of scaffolds is influenced by pore homogeneity. Li *et al.* [34] showed lower compressive strength of NiTi scaffolds processed with a non-sieved space holder, as compared with that prepared using a sieved space holder. In this case, the non-sieved space holder led to non-uniform and irregularly shaped macro-pores in scaffolds. As a consequence, these scaffolds suffered from severe stress concentrations and collapsed at low stress levels. In addition, it was difficult to obtain a linear correlation between the strength and porosity of scaffolds processed with the non-sieved space holder [34]. Using the finite element method (FEM), Niu *et al.* [80] confirmed this finding; the relationship between elastic modulus and pore distribution in scaffolds with a random pore distribution could not be predicted, in contrast to scaffolds with a pore distribution in a regular array.

### 3.2. Effect of Mixing on Segregation

**Figure 6.**
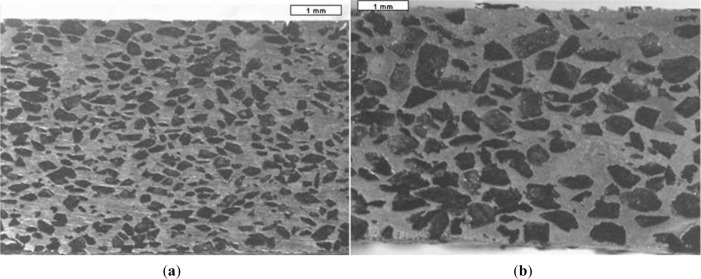
Sintered pure titanium scaffolds having 60% porosity processed with space holders having size ranges of (**a**) 250–500 μm and (**b**) 500–1000 μm. Reprinted with permission from [79]. Copyright 2011, Maney Publishing.

Inhomogeneous distribution of macro-pores in scaffolds, as well as inhomogeneous distribution of space-holding particles in scaffold preforms, is often attributed to powder segregation that occurs during the mixing process. Segregation, or the separation of mixed powder components, occurs due to the differences in size and density between powder components. Two modes of powder segregation have been recognized, *i.e.*, buoyancy and percolation [81,82]. In the buoyancy mode, powder segregation occurs as a result of the difference in powder density; heavier particles sink to a lower level of the mixture, while lighter ones rise up. Powder segregation with the percolation mode occurs because of the differences in particle size and size distribution; smaller particles tend to fall through the interstices of larger particles and settle at the bed of a mixing container. During the mixing of metallic matrix powder with space holder, segregation in the buoyancy mode and in the percolation mode may simultaneously occur as both smaller but heavier metallic matrix particles and larger but lighter space holding particles are involved in the mixing system. A segregated powder mixture yields clusters of pores in certain regions of the scaffolds, after the removal of the space holder (see Figure 6) [79]. It has been reported that matrix particles should be approximately several times smaller than space-holding particles in order to improve the sinterability of metal matrix powder [50] (see Section 6). It has however also been reported that uniform distribution of pores in titanium scaffolds can be achieved by mixing titanium matrix particles with an average size of 45 μm and a carbamide space holder with an average size of about 51 μm, instead of >223 μm [83].

Segregation occurring during the mixing of metal matrix powder and space holding particles can be minimized by using binders. With a binder added to the mixture, granular materials are formed, composed of space-holding particles coated with smaller metal matrix particles [50,52,66,84]. Such granular materials can then be compacted to form a green body. Binders are mostly prepared in the liquid form and have to be selected appropriately to avoid contamination due to harmful elements possibly retained in the final scaffold products. The criteria for appropriate binders have been established, based on their biocompatibility and non-toxic properties. In addition, binders must be able to produce an adequate binding strength between metal matrix powder and space-holding particles and not be reactive to both of the powders. Water, for example, may be an inappropriate binder for some water-soluble space holders, such as carbamide, ammonium hydrogen carbonate and sodium chloride, as it reacts with these powders and consequently distorts the sizes and shape of space-holding particles. Up till now, a number of binders have been used for the fabrication of metallic scaffolds, such as polyvinyl-alcohol (PVA) [41,42,52,59,67,69,74,75,85], polyethylene-glycol (PEG) [28], polymethyl metacrylate (PMMA) [66], paraffin [44,84,86] and ethanol [39,46,65,86,87]. A multi-component binder consisting of high-density poly-ethylene (HDPE), paraffin wax, poly-ethylene glycol and stearic acid has also been used in the fabrication of scaffolds through powder injection molding [88,89]. In addition, binders have been used to increase the green density of scaffold preforms. Detailed information on the green density of scaffold preform can be found in Section 4. Jha *et al.* [74] showed a higher green density of titanium scaffold preforms (1.38 g·cm^−3^) compared to the value from theoretical calculation (1.12 g·cm^−3^), due to the addition of PVA binder. The amount and concentration of binder are of critical importance and should be chosen appropriately so as to optimize the mixing process and obtain a homogeneous mixture of metal matrix powder and space-holding particles. Despite the importance, the influence of binder parameters on the properties of the green body as well as on the space holder distribution in the mixture is rarely discussed in the open literature.

Table 3 summarizes the reports on the mixing process for metal matrix powder and space-holding particles in the preparation of metallic biomedical scaffolds. Besides binders, mixer type and mixing duration are important factors that may affect the results of the mixing process.

Obviously, mixing of metal matrix powder and space holding particles is a step of critical importance in scaffold fabrication. Any failure in the mixing process, leading to an inhomogeneous distribution of space holder particles and consequently an inhomogeneous distribution of macro-pores in scaffolds cannot be repaired at the subsequent steps of scaffold fabrication.

## 4. Compaction

### 4.1. Effect of Compaction on Powder Particles

Compaction is performed after mixing to achieve a certain green strength that can keep the mixture of metal matrix powder and space holding particles intact during the subsequent steps of scaffold fabrication, *i.e.*, space holder removal and sintering. During compaction, granular materials obtained from mixing are densified, forming the green body of scaffolds or scaffold preforms. Stages involved in the powder compaction process have been well described in the literature. Before compaction, loose powder particles or granular materials have no bonding strength, except for a small area of inter-particle contact. A large number of voids are present in the interstices of loose powder particles or granular materials. When compaction begins, powder particles or granular materials rearrange themselves, fill the voids and increase packing coordination. As compacting pressure increases, the number of contact points and the contact area of granular materials increase, leading to densification and the formation of a composite green body made of metal matrix powder and space holding particles.

### 4.2. Effect of Compaction on Green Density and Green Strength

Powder compaction improves the sinterability of metallic powders. Effective metallurgical bonding between metallic powder particles can be achieved, only when there is no oxide film on the powder particle surface [48,86]. During compaction, surface oxide film may be disrupted, allowing direct contact of compacted powder particles. The disruption of oxide film by compaction occurs due to large shear strains, stress concentrations, scratching and jabbing that occur when metallic powder and space holding particles are pressed against one another under a given compacting pressure [86].

**Figure 7.**
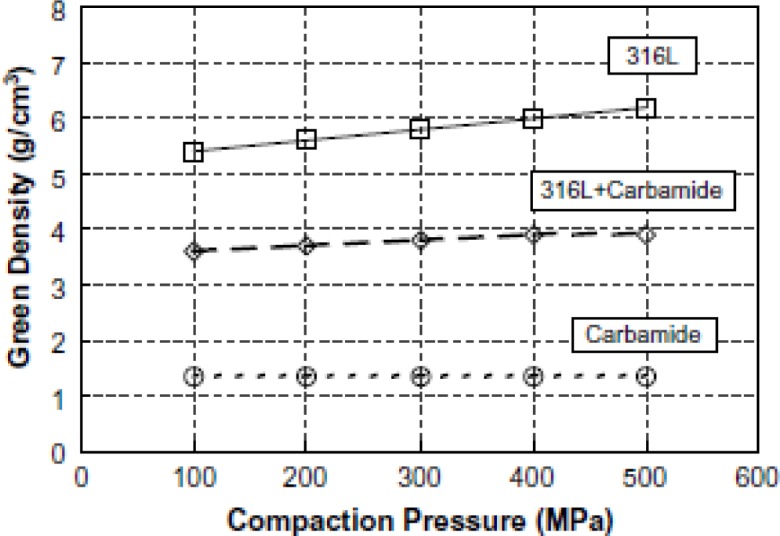
Effect of compaction pressure on the green strength of powder compact. Reprinted with permission from [66]. Copyright 2006 Elsevier.

Scaffold preforms prepared with powder compaction are often evaluated in terms of green density ρ_g_, as mathematically expressed in Equation (3),

$$\rho_{g}=m_{g}/V_{g}$$ (3)

where *m*_g_ and *V*_g_ are the mass and volume of the scaffold preform. The green density of the scaffold preform increases with rising compacting pressure applied to the compact [46,66,87]. However, the presence of less-densified space-holding particles than metal matrix powder particles results in a less significant increase in the green density of scaffold preform. Bakan showed in Figure 7 that an increase in compacting pressure from 100 to 500 MPa could considerably enhance the green density of 316L stainless steel powder [66]. Densification of stainless steel powder mixed with 70 vol% carbamide was however less significant than that of the powder without carbamide under the same compacting pressure. The green density of carbamide powder compacts was found to be independent of compacting pressure applied [66]. Similarly, the green densities of titanium and Ti-6Al-4V scaffolds were found to increase with increasing compacting pressure [31,46,87]. Moreover, it was observed that the density of scaffold preforms decreased with increasing volume fraction of carbamide space-holding particles [87]. However, the relationship between the green density of scaffold preforms and the sizes of space-holding particles was unclear [87].

The problems encountered in the compaction of granular materials in the preparation of metallic scaffolds have been recognized in the literature, such as (i) low structural integrity of scaffold perform; (ii) deformation and then breakage of space-holding particles and (iii) inhomogeneous pressure distribution in the compact.

Low green strength is not desired, as it may result in the collapse of scaffold preforms at the subsequent processing steps. Torres *et al.* [35] reported collapsed titanium matrix particles during the water leaching process for the removal of space-holding particles. Titanium scaffold preforms processed under compacting pressures lower than 200 MPa could not remain intact after the removal of NaCl space-holding particles through water leaching [35]. The ductility of powder material also influences the structural integrity of scaffold preforms. Ductile powder materials usually correspond to more stable green compacts than brittle ones, either before or after the removal of space-holding particles. Binder is therefore often used to improve the green compact stability of brittle powder materials [50].

**Figure 8.**
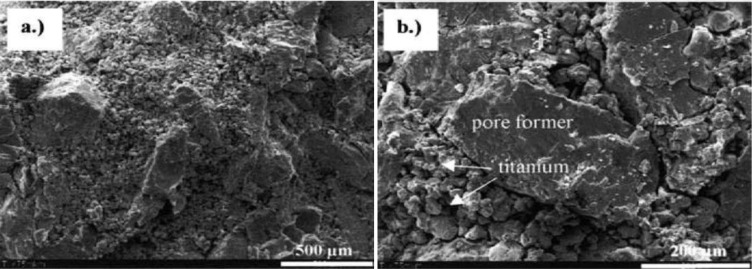
Breakage of space holder particles (as pore former) due to compaction. (**a**) fracture surface of titanium scaffold preform prepared with 70 vol% space holder and processed with compaction pressure of 350 MPa; (**b**) fractured space holder (pore former) surrounded by titanium particles. Reprinted with permission from [33]. Copyright 2004 Maney Publishing.

Deformation and then breakage of space-holding particles in scaffold preforms occur when their critical stresses to fracture are exceeded by the compacting pressures transferred locally to these particles [33,72]. Fracture of ammonium hydrogen carbonate particles in a mixture with titanium matrix particles under a compacting pressure of 350 MPa has been reported [33]. Under a given compacting pressure, space-holding particles serve as the bridges that separate metal matrix particles. With increasing compacting pressure, metal matrix particles tend to press space-holding particles more strongly. Once their elastic limit and critical strength are exceeded, space-holding particles are deformed and broken (see Figure 8). Deformation of magnesium and carbamide space-holding particles during compaction has been reported [29,72,86]. Deformation distorted the resulting macro-pore sizes and morphology and consequently induced anisotropic properties of scaffolds [72]. Furthermore, there was a tendency that broken space-holding particles were trapped in scaffold preforms and could not be completely removed through water leaching [35]. Since compaction on the one hand enhances the strength of compacts it may on the other hand distort space holder geometry, attempts have been made to determine an optimum compacting pressure that balances these opposite results. An optimum compacting pressure can be determined, based on experimental results and theoretical calculation.

### 4.3. Determination of Optimum Compacting Pressure

Several experimental techniques have been used to determine an optimum compacting pressure for scaffold fabrication with the space holder method, such as (i) visual inspection; (ii) microhardness test; (iii) compression test and (iv) shrinkage evaluation. In visual inspection, direct observation of space-holding particles is made after compaction. Bakan showed that compaction under pressures higher than 100 MPa could break carbamide space-holding particles that were embedded in 316L stainless steel matrix powder [66]. Smorygo *et al.* [29] showed elliptical pores in titanium scaffolds as a result of distorted carbamide space-holding particles after compaction at a pressure of 500 MPa. Gligor *et al.* [38] established 400 MPa as an optimum compacting pressure to achieve sufficient interparticle bonding strength without deforming space-holding particles. Kotan and Bor [30] reported that the structural integrity of Ti-6Al-4V scaffold preforms could not be maintained after compaction at pressures below 300 MPa and hence a compacting pressure of 450 MPa was used in their research. With the microhardness method, an optimum compacting pressure can be determined, based on the uniformity of cell wall microhardness in scaffolds. For example, compaction at a pressure of 250 MPa resulted in titanium scaffold cell walls with uniform microhardness [31]. In addition, the determination of an optimum compacting pressure, based on compressive yield strength and shrinkage, was carried out by Niu *et al.* [90]. With this method, the maximum compressive yield strength and minimum shrinkage of sintered titanium scaffolds were achieved when a compacting pressure of 200 MPa was applied in preparing scaffolds. Examples of the experimental ways of determining optimum compacting pressures are summarized in Table 4.

The determination of an optimum compacting pressure from theoretical calculation has only been reported in a limited number of papers. Jha *et al.* [74] and Mondal *et al.* [91] introduced the rule of mixture as expressed in Equation (4) to estimate an optimum pressure σ for uniaxial die compaction of titanium powder and NaCl space-holding particles:

$$\sigma =x{\bar{\sigma }}_{\text{sh}}+\left(1-x\right){\bar{\sigma }}_{m}$$ (4)

where ${\bar{\sigma }}_{\text{sh}}$ and ${\bar{\sigma }}_{\text{m}}$ are the strengths of space-holding and metal matrix powder materials, respectively and x is the volume fraction of the space holder in the mixture. In applying this equation, the applied pressure was assumed to be shared proportionally according to the volume fractions of metal matrix powder and space-holding particles. For instance, in order to produce a titanium scaffold with 80% porosity, a mixture of titanium matrix powder and NaCl space holding particles has to be prepared with a volume fraction of space holder being 0.8. Since the strengths of titanium powder material and NaCl are 450 and 60 MPa, respectively, the optimum compacting pressure determined using Equation 3 is 138 MPa [74]. In practice, compacting pressure applied to granular materials must be higher than this value in order to overcome inter-particle frictional forces and the friction between powder particles and die wall.

### 4.4. Effect of Compaction on Porosity

An inhomogeneous pressure distribution over powder compacts may lead to (i) the variation of green density in the scaffold perform; (ii) an inhomogeneous distribution of space-holding particles as well as an inhomogeneous distribution of resultant macro-pores in the scaffold and (iii) deteriorated mechanical properties of the scaffold [31,36,79,92]. Detailed information on the variations in green density and green strength of scaffold preforms as a result of not optimum compaction is discussed later in this communication.

Compaction influences the porosity of scaffolds. Zhao *et al.* [36] reported reductions in the total porosity of porous NiTi alloy scaffolds with increasing compacting pressure and increases in porosity with increasing volume fraction of NaCl space holder [36]. Since the total porosity of the scaffold is calculated from the sum of macro-porosity and micro-porosity, this finding may indicate that micro-porosity decreases with increasing compacting pressure due to increasing packing coordination (interparticle contact area) in the scaffold [31,42]. Similarly, Esen and Bor [42] revealed lower porosity levels in Ti-6Al-4V scaffolds from compacted and then sintered powder, in comparison with sintered loose matrix powder. This phenomenon was confirmed in other studies on the removal of space-holding particles. Torres *et al.* [35] reported that the lowest compacting pressure over a range of 200–800 MPa resulted in the highest porosity level in titanium scaffold preforms. As a consequence, the time needed for NaCl removal through water leaching became shorter. Bekoz and Oktay [52] showed that water leaching of carbamide particles ran more slowly through the green body of stainless steel foams that were compacted at a pressure greater than 200 MPa, confirming a reduced number of micro-pores in scaffold preforms. Furthermore, Li *et al.* [31] showed a decreased sintering index of titanium scaffolds with increasing compacting pressure. Since an increase in the sintering index means an increase in the shrinkage level of scaffolds during sintering, this finding implies a reduction in micro-porosity by compaction.

The effect of compaction on the macro-porosity of scaffolds has been reported. As discussed earlier, deformation and fracture of space-holding particles that may occur during compaction will distort the sizes and shape of macro-pores in the resulting scaffolds [72]. On the other hand, macro-porosity of scaffolds is unaffected by compaction, since the content of space holder remains unchanged [35]. However, pore interconnections increase with increasing compacting pressure because of the deformation and coalescence of space-holding particles that form interconnected pores in the end [93].

### 4.5. Common Compaction Techniques

Several techniques of compaction have been used in the fabrication of metallic scaffolds, *i.e.*, (i) uniaxial die compaction [33,41,42]; (ii) isostatic compaction [49,55] and (iii) injection molding [40,88,94]. Furthermore, the compaction of metal matrix powder and space-holding particles has been conducted either at room temperature (cold compaction) [31,33,74] or elevated temperatures (hot pressing) [55,72].

**Figure 9.**
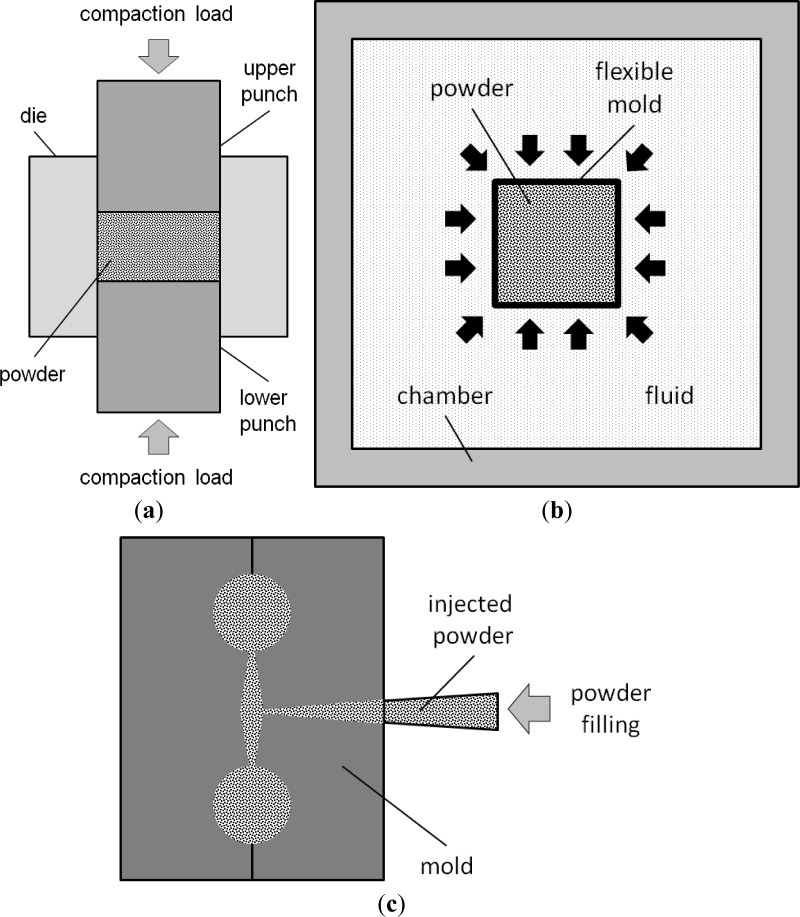
Common techniques of compaction in the fabrication of metallic scaffolds with the space holder method: (**a**) uniaxial die pressing; (**b**) isostatic pressing and (**c**) injection molding.

As shown in Figure 9a, uniaxial die compaction is performed with the aid of a pair of punches that move uniaxially through a die filled with powders or granular materials. Once powders are loaded in the die, the upper punch compresses the powders or granular materials. The lower punch supports the compressed powders or granular materials and delivers reaction against the upper punch. Compacted powders or granular materials are then ejected from the die, once compaction is accomplished. Limitations of uniaxial die compaction as a result of (i) inter-particle friction and (ii) the friction between powder particles and die wall have been recognized. These limitations are responsible for the variations in pressure and green density in powder compacts. It is reported that in single-action die compaction, the highest compacting pressure is experienced by the powder particles that are located in the circumference of the cylindrical compact nearby the acting punch. As a consequence, the highest green density of the compact is achieved in this location. On the other hand, the lowest compacting pressure that yields the lowest green density is found in the circumference of the lower part of the compact [92]. With increasing aspect ratio or height-to-diameter ratio of compacts, the variation in green density in powder compacts becomes more pronounced. Since green strength corresponds proportionally to green density, the powder particles located in the region with the lowest green density are prone to collapse.

The variations in compacting pressure and green density in uniaxially compacted powder influence the porous architecture of metallic scaffolds. Zhao *et al.* [36] showed that the variation in compacting pressure led to the increases in the inhomogeneity of pore distribution in NiTi scaffolds. Li *et al.* [31] showed the variation in cell wall or framework microhardness in titanium scaffolds, depending on the compacting pressure applied. In their study, the cell wall microhardness of titanium scaffolds processed under a compacting pressure of 150 MPa increased linearly from the center to the edge of scaffolds. This could be attributed to the friction between titanium powder particles and the die wall surface, which resulted in a hardening effect on these particles in the vicinity of the die wall. Such a die wall effect became less significant with increasing compacting pressure until 250 MPa, as indicated by uniform cell wall microhardness in scaffolds. However, the cell wall microhardness distribution in scaffolds became less homogeneous, as increasing packing coordination and inter-particle friction became dominant when compacting pressure was increased to 350 MPa. Attempts to minimize the variations in pressure and green density in scaffold preforms processed with uniaxial presses have been made by introducing double-action die compaction [41,42,66,69]. With this method, powders or granular materials are compacted from both sides, using two moving punches from the ends of the die. As a consequence, more uniform densification is achieved over cylindrical powder compacts.

A homogeneous distribution of green density in scaffold preforms can also be achieved with isostatic powder compaction. In this method, a flexible mold containing powders or granular materials is pressurized with fluid, e.g., water or oil, to generate a powder compact (see Figure 9b). Isostatic compaction can also be used for preparing powder compacts with complex shapes.

Injection molding is another compaction technique that allows mass production of scaffolds with complex shapes at low production costs [88,89]. In principle, this technique combines the versatile plastic injection molding and conventional powder metallurgy technique for the fabrication of parts from powders (see Figure 9c). With this technique, feedstock composed of metal matrix powder and space holding particles is injected into a mold.

## 5. Space Holder Removal

Macro-pores in metallic scaffolds prepared with the space holder method are formed, after the removal of space-holding particles. In other words, the removal process determines the geometry of macro-pores, as well as the structural integrity and purity of scaffolds. Complete removal of space-holding particles is desired in order to obtain the requisite scaffold porosity and to prevent scaffolds from contamination by residual space-holding particles [75]. It has been reported that the resulting scaffold porosity is often found to deviate from the designed value due to entrapped space holder residues in scaffold preforms and the collapse of matrix particles during the removal of space-holding particles. In addition, the low structural integrity of scaffold preforms after the removal of space holder also causes the distortions of macro-pore geometry and difficulties in handling scaffold preforms prior to sintering [33,35,39]. All these problems indicate the importance of this fabrication step for the quality of the final scaffolds. Currently, two techniques for space holder removal are commonly used, *i.e.*, (i) heat treatment and (ii) leaching in liquid.

### 5.1. Space-Holder Removal through Heat Treatment

Space holder removal by means of heat treatment is conducted, based on the thermal decomposition and evaporation of space-holding material. Table 5 shows a number of common space holders and their decomposition and removal temperatures.

As shown in Table 5, the removal of space holder takes place at a temperature higher than the decomposition temperature of this particular space holder material. At the removal temperature, the space holder evaporates and escape from scaffold preforms along with flushing gas that leaves the furnace [30,34]. The removal temperature where a space holding material can be eliminated completely is often determined from thermal analysis.

**Figure 10.**
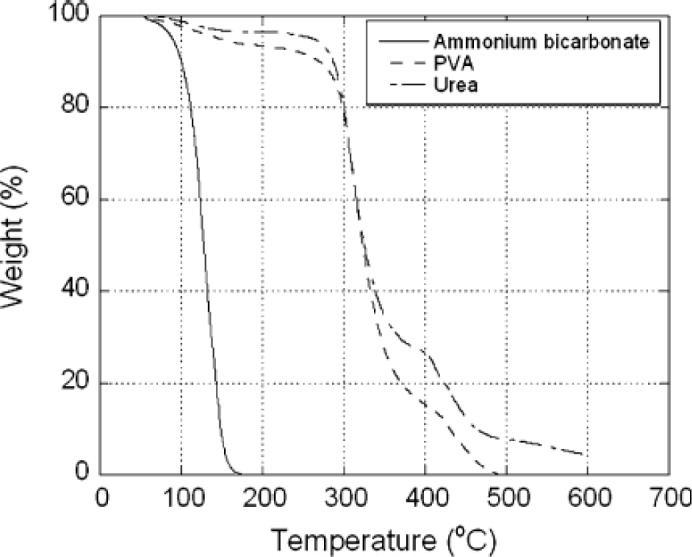
Thermo-gravimetric analysis (TGA) of space holder and binder materials. Reprinted with permission from [75]. Copyright 2009, Springer.

Both the decomposition and removal temperatures of a particular space holder material can be determined by means of thermo-gravimetric analysis (TGA) [33,75]. Figure 10 shows the results obtained from TGA, revealing weight losses due to the escape of ammonium hydrogen carbonate and carbamide that were used as the space holder in the study of Dizlek and his co-workers [75]. Decomposition of ammonium hydrogen carbonate (or ammonium bicarbonate) started at about 50 °C and the reaction continued until this space holder material was completely removed at 175 °C. Weight reduction of another space holder, carbamide or urea, started at a temperature above 200 °C but it could not be removed completely until 600 °C. In addition, complete removal of binder material, polyvinyl-alcohol solution (PVA), occurred at about 480 °C. Similarly, thermo-gravimetric analysis conducted by Laptev and his co-workers [33] showed that the weight loss of ammonium hydrogen carbonate upon heating started at a temperature of 60–80 °C and proceeded more intensively as a result of water evaporation when the temperature increased to 100 °C and higher. The weight of ammonium hydrogen carbonate decreased rapidly, resulting in its complete removal at a temperature higher than 150 °C. Based on these results, the removal of ammonium hydrogen carbonate from scaffold preforms was recommended to be carried out at 150 °C.

In practice, the removal of space holder material from scaffold preforms runs more slowly during the real process than during TGA analysis [33]. This can be attributed to the time required for the diffusion of removal products through tortuous channels in the interior of scaffold preforms. In order to facilitate the total elimination of space holder, dwelling at a removal temperature for several hours [33], ranging from 1–21 h [28,34,45,46,79,87], is needed. Macro-pore interconnections in scaffold preforms contribute to speeding up the removal process, *i.e.*, by providing access for the diffusion of removal products. With more interconnected macro-pores in scaffold preforms, space holder removal can be completed within a shorter time [93]. After the process, macro-pores are formed in scaffold preforms with a morphology replicating space-holding particles [34] and surrounded by matrix particles [33].

There are two problems recognized in association with space holder removal through heat treatment, *i.e.*, (i) low green strength and (ii) contamination of the porous scaffold preforms. Laptev *et al.* [33] indicated that after the removal of space-holding particles, scaffold preforms were very weak, because green strength relied only on the mechanical interlocking of irregularly shaped titanium particles. To prevent porous scaffold preforms from collapsing, they should be handled with care during transporting and sintering.

Contamination to scaffolds by the decomposition products of space-holding particles has been reported. Reactions between titanium matrix powder and decomposition products of space-holding particles may occur at temperatures between 300 and 600 °C and result in a detrimental effect on the ductility of titanium scaffolds due to the increases in carbon (C), oxygen (O) and nitrogen (N) contents in scaffolds [75]. To prevent scaffolds from contamination, the removal of space-holding particles was conducted in vacuum or under flushing argon gas [34,41,42,46,87]. For instance, thermal removal of carbamide space-holding particles was carried out in vacuum [46,87] to prevent the formation of unacceptable biuret in scaffold products [33]. On the other hand, heat treatment for the removal of ammonium hydrogen carbonate can be performed in atmospheric air without inducing contamination [33,79]. The use of biocompatible, food-grade particles such as magnesium and tapioca starch has been recognized as an alternative way to preventing contamination [37,41,42,67,69].

### 5.2. Space-Holder Removal through Leaching

The removal of space-holding particles from scaffold preforms with liquid leaching is performed, based on the dissolution of space holder material in certain liquids. In this method, scaffold preforms are immersed in liquid to allow the dissolution and leaching of space-holding particles from compacts. This technique is often preferred, because of its low environmental impact, compared with the heat treatment discussed above [66]. Water has been chosen as a leaching medium for many space-holders, such as carbamide [29,52,66,86,95], sodium chloride [35,36,55], saccharose [39] and corn starch dextrin [38]. However, other leaching media such as NaOH [86], hydrochloric acid [72], acetic acid [43] and ethanol [72] have also been used, mainly for the removal of space holders that are not dissolvable in water, such as magnesium [72] and stainless steel [43]. Table 6 shows a few examples of space holders and solvents for leaching.

In principle, leaching of space-holding particles from scaffold preforms is similar to the solvent debinding process for injection-molded metallic parts [76,96,97]. Dissolution and diffusion are the main mechanisms that govern the leaching process. Once scaffold preforms are immersed in a solvent, space-holding particles at the surface dissolve immediately, forming openings and interconnected pores that allow the liquid to penetrate into the interior of scaffold preforms. The removal process is then governed by the simultaneous dissolution of space-holding particles in the interior of scaffold preforms and by the diffusion of fresh solvent and dissolution products through macro-pore interconnections [76].

The solubility of space holder material in a particulate solvent is considered of critical importance, determining the resulting structure of scaffold preforms after leaching. The structural integrity of scaffold preforms could be deteriorated and the matrix framework of scaffolds could be oxidized due to a too long immersion time [35,86]. On the other hand, a short leaching time may lead to incomplete removal of space holder and consequently contamination to scaffold preforms. A highly soluble space holder material in a leaching medium is therefore a preferred choice to ensure a quick dissolution process and complete removal of space-holding particles. In addition, the leaching medium should be chosen appropriately. A solvent with a low concentration of HCl, for example, could induce cracks in the resulting porous titanium scaffold preforms, while an excessive HCl concentration could chemically deteriorate scaffold preforms [72].

**Figure 11.**
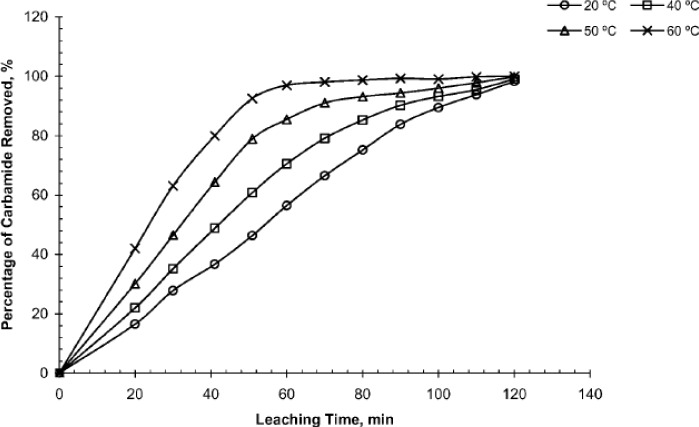
Effect of water temperature on the removal of space holder by leaching. Reprinted with permission from [95]. Copyright 2008, Maney Publishing.

Leaching rate of space-holding particles in a given solvent increases with increasing space holder content in scaffolds [35]. With a greater space holder content, more interconnected macro-pores may be formed, providing greater access for the diffusion of dissolved products from the interior of scaffold preforms. Leaching rate is also enhanced by micro-pores that are formed in the matrix framework of scaffolds [35] and the shape of space-holding particles. Bekoz and Oktay [52] showed that immersion in water for 150 min could remove 97% of irregularly shaped carbamide particles from scaffold preforms. However, a longer time was required to remove 93% of spherical carbamide particles from scaffold preforms [52]. This finding indicates that in scaffold preforms prepared from irregular carbamide particles, the number of isolated space-holding particles was reduced, leading to faster removal of these particles [52]. Recently, the leaching process was conducted in water and other liquid media at elevated temperatures to increase the dissolution rate of space-holding particles [35,38,39,95]. Torres *et al.* [35] reported faster dissolution of NaCl space holder in water at 50–60 °C than at room temperature. Gulsoy and German also showed that the removal rate of carbamide space holder increased with water temperature (see Figure 11) [95]. Furthermore, Gligor *et al.* and Jakubowicz *et al.* [38,39] performed water leaching at 80 °C with magnetic stirring to speed up the leaching of corn starch and sugar particles from titanium scaffold preforms, respectively. Moreover, the use of agitated liquid medium [35,39] and electrolytic process [43] has been reported to increase the removal rate during leaching. To facilitate the removal of steel space holder through leaching, Kwok *et al.* [43] utilized an electrolytic cell in a 10 vol% acetic acid aqueous solution with titanium sheet and titanium-steel compact as the cathode and anode, respectively.

**Figure 12.**
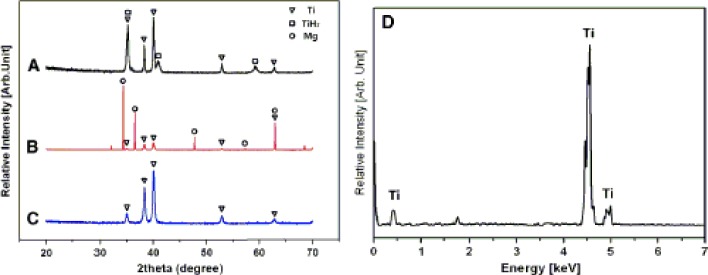
X-ray diffraction (XRD) peaks: (**A**) for porous green body after Mg removal; (**B**) for Ti/Mg compact; (**C**) for compact packed only with Ti powder; (**D**) energy-dispersive X-ray spectroscopy (EDS) peaks for porous green body after Mg removal (initial Mg content of 60 vol%). Reprinted with permission from [72]. Copyright 2013, Elsevier.

Contamination to scaffold framework in association with residual space-holding particles after the leaching process is rarely reported. Using X-ray diffraction (XRD) and energy-dispersive X-ray spectroscopy (EDS) analysis (see Figure 12), Kim *et al.* [72] showed no peaks of magnesium, which indicated no residuals from magnesium space holder in porous titanium framework after the leaching process. In this study, magnesium space-holding particles were removed from titanium scaffold preforms by dipping alternatively in 2 N hydrochloric acid (HCl) and ethanol for 24 h. After leaching, porous scaffold preforms were cleaned with ethanol and dried in an oven at 60 °C. Peaks that indicate the presence of TiH_2_ were however seen in the porous titanium framework due to its reactions with the HCl solvent. Nevertheless, these peaks disappeared after sintering, as a result of the transformation of TiH_2_ to pure titanium through thermal decomposition.

## 6. Sintering

Sintering is performed at high temperatures where bonding between metal matrix particles in scaffold preforms takes place. Bonded matrix particles build up the framework of the porous structure of scaffolds. Through sintering, the final structure of metallic scaffolds can be achieved. Stages involved in the sintering of metallic powders are clearly described in the literature [45]. At the beginning, inter-particle bonds and necks are formed at powder particle contact points. Atoms of powder particles are thermally activated, leading to mass diffusion and neck growth at inter-particle contact points. As sintering proceeds, voids at powder particle interstices are rounded, along with densification and grain growth that occur simultaneously. Towards the end of sintering, powder densification keeps occurring but at a slower rate than the earlier stage of the densification process.

### 6.1. Effect of Sintering Process Parameters on Densification and Porosity

Densification of matrix powder during sintering leads to the increases in the microhardness of the scaffold cell wall [31]. As a consequence, the mechanical properties of scaffolds are enhanced. Incomplete sintering, as a result of insufficient diffusion in the inter-particle contact area, leads to the formation of micro-pores [30]. Micro-pores are not desired for the mechanical properties of scaffolds, as they reduce the load-bearing cross-sectional area of cell wall and consequently deteriorate the compressive strength of scaffolds [28,30,84]. However, several researchers argued that the presence of micro-pores with sizes ranging from 5 to 20 μm could increase the total porosity and osteoinductvity of scaffolds [41,45].

Micro-porosity of sintered scaffolds can be controlled by adjusting the parameters applied to the sintering process, such as (i) temperature [23,42,75], (ii) time [31,83] and (iii) pressure [23]. Oh *et al.* [23] reported that the porosity of sintered titanium scaffolds decreased as sintering temperature increased from 900 to 1300 °C. Similarly, the porosity of Ti-6Al-4V scaffolds prepared from loose powder decreased linearly with increasing sintering temperature [42]. This finding corresponds to the increases in the number of inter-particle contacts and enhanced growth of sintering necks that eventually lead to densification and the reduction of micro-pore sizes as sintering temperature increases [75]. A too high sintering temperature is however not preferred, as it may lead to the evaporation of certain alloying elements in the matrix powder and induce excessive partial melting [87]. Densification of metal matrix powders in scaffolds also occurs with increasing sintering time. To indicate enhanced densification of matrix powder with prolonged sintering time, Li *et al.* and Sharma *et al.* [31,83] showed significant increases in microhardness at the scaffold cell wall and reductions in pore size, as sintering time was extended. Scaffold porosity also decreased, due to the increases in inter-particle contact, if pressure was applied to scaffold preforms during the sintering process (pressure-assisted sintering) [23].

Macro-pores in sintered scaffolds are formed from the space occupied by removed space-holding particles. Using the space holder method, scaffold porosity increases to a certain level controlled by the volume fraction of the space holder added to the metal matrix powder [79,86]. Although a number of reports claimed that macro-pores present in sintered scaffolds were similar to space-holding particles [38,86], the porous structure of the resulting scaffolds was indeed rather difficult to control. Wang *et al.* [45] reported the deviations of macro-pore sizes in sintered porous Ti-Nb-Zr alloy scaffolds, *i.e.* 300–800 μm, from the initial sizes of space-holding particles used in scaffold fabrication, *i.e*. 500–800 μm. Bram *et al.* and Li *et al.* [34,50] showed smaller macro-pores in sintered scaffolds than the sizes of the space holder particles used in scaffold processing, although this reduction was insignificant [50]. In addition, macro-pore surfaces of sintered scaffolds were rough, containing micro-pores and did not reflect the surfaces of space-holding particles [59,86]. This characteristic could be observed in scaffolds prepared with small matrix powder particles [38]. Figure 2 confirms the presence of rough inner surfaces of macro-pores in sintered titanium powders. However, Bram *et al.* [50] indicated that the use of small matrix powder particles could lead to fully densified framework without micro-pores.

As discussed earlier, the presence of micro-pores leads to the deviations in the porosity of sintered scaffolds from the designed value. With the space holder method, micro-pores can hardly be avoided. The number of micro-pores can however be reduced by increasing space holder content. With a greater space holder content, the thickness of scaffold framework or cell wall decreases [67,74], limiting the chance of micro-pore formation in the framework. Accordingly, the difference between the resulting scaffold porosity level and the content of space holder added to the mixture decreases [35]. Aydogmus *et al.* [67] showed decreases in micro-porosity down to 1% with an addition of 80 vol% magnesium space-holding particles. On the other hand, Smorygo *et al.* and Amigo *et al.* [29,79] argued that both total porosity and interconnected porosity were lower than the space holder content mixed with matrix powder, due to the shrinkage of metallic framework during sintering.

The shrinkage of powder compact during sintering occurs as a result of inter-particle neck growth and mass diffusion that lead to the elimination of micro-pores and densification [33,67]. Laptev *et al.* [33] noticed axial and radial shrinkages of cylindrical titanium scaffolds prepared with 0–70 vol% space holder contents and with 100–450 MPa compaction pressures by 95%–14% and 10%–15%, respectively, after sintering at temperatures of 1200–1300 °C for 1–3 h. Similarly, Aydogmus and Bor [67] revealed the shrinkages of NiTi scaffolds processed with 80 vol% magnesium space holder by <2.5% in height and <3.5% in diameter after sintering at 1100 °C for 1 h. Shrinkage has to be controlled; otherwise it may ruin the porous structure of scaffolds. In addition to the control of sintering temperature and time [33], the shrinkage of scaffolds can be controlled through compaction prior to sintering [33] and the addition of a specific amount of space holder [36]. A higher green density produced by compaction reduces the shrinkage of sintered scaffolds. Since space-holding particles reduce green density, shrinkage increases with increasing space holder content [33]. Space-holding particles serve as the bridges between matrix particles, which obstruct the effective pressure transmission to matrix particles during compaction. Esen and Bor [41] showed that a critical volume fraction of magnesium space holder that could be mixed with matrix particles was limited to 55%–60%, above which compacts shrank significantly during sintering. With a lower magnesium content, shrinkage was insignificant, but porosity was higher than the volume fraction of magnesium added, due to the formation of micro-pores in scaffold framework [41]. However, shrinkage rate decreased in high-porosity scaffolds, as a result of thinner cell wall subjected to densification [31]. It has also been reported that cell reorientation occurs due to shrinkage during sintering. Jha *et al.* [74] showed the formation of nearly spherical cells having an aspect ratio of 0.98 in sintered titanium scaffolds processed with cubical NaCl space-holding particles.

Sintering may be performed in a single cycle together with heat treatment for the thermal removal of space-holding particles [18,25,37,59]. Mansourighasri *et al.* [37] performed heat treatment at 450 °C for 2 h to remove tapioca starch from titanium scaffold preforms, followed by sintering at a temperature of 1200 °C for 3 h. Additional thermal processing may also be conducted to ensure complete removal of residues from scaffold preforms. A heat treatment at 850 °C for 1 h was conducted by Bhattarai *et al.* [46] to allow surface oxides, water molecules and contaminants to volatilize off from scaffold preforms. Sintering was then performed by raising furnace temperature to 1200 °C and holding scaffold preforms at this temperature for 2 h. Sintering may also be performed in a separate furnace with which space holder removal is performed. Amigo *et al.* [79] conducted heat treatment at a temperature of 80 °C for 21 h in atmospheric air to remove ammonium hydrogen carbonate space holder before sintering. Sintering was then performed in a vacuum furnace at 1300 °C for 2 h.

### 6.2. Contamination Induced during Sintering

**Figure 13.**
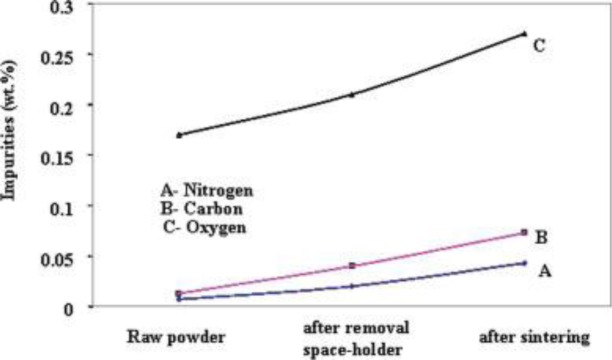
Chemical analyses of oxygen, carbon and nitrogen contents in Ti-6Al-4V parts after different processing steps. Reprinted with permission from [46]. Copyright 2008, Wiley Periodicals.

Contamination due to sintering is critical, as it potentially deteriorates the properties of metallic scaffolds. Contamination of titanium scaffolds during sintering can be induced by (i) residual space holder in the scaffold perform; (ii) contaminants in the sintering furnace and (iii) exposure to atmospheric air during sintering. Reactions between residual space-holding particles and the matrix framework of scaffolds during sintering, lead to increased unacceptable impurities in scaffolds [38,39,91]. Impurities in sintered scaffolds also increase due to residual contaminants in the sintering furnace that may react with scaffold framework during sintering. As shown in Figure 13, Bhattarai *et al.* [46] noticed no significant increases in C, N and O contents after the removal of carbamide space-holding particles from Ti-6Al-4V scaffolds. However, the concentrations of C, N and O increased slightly after sintering. As the sintering process was carried out in vacuum (2 × 10^−3^ torr), this might indicate that the increased concentrations of C, N and O impurities in sintered scaffolds could be attributed to the contaminants that were present in the furnace used [46]. A number of studies on titanium scaffold contamination due to sintering have been conducted. Despite its excellent mechanical properties and biocompatibility, titanium has extreme affinity to oxygen and nitrogen when processed at high temperatures. Oxygen and nitrogen can dissolve rapidly in titanium at temperatures above 400 °C [20]. Kim *et al.* [72] showed an increased oxygen content from 0.297 to 1.118 wt% in titanium scaffolds after sintering. Sintered titanium exhibits brittle behavior, once the oxygen content exceeds a critical level for retaining ductility [72]. Therefore, attempts have been made to prevent titanium scaffolds from contamination during sintering by, for instance, introducing a vacuum environment [37,46] and flushing argon gas [41,42] into the sintering furnace.

The importance of controlled sintering atmosphere was also noticed in a study conducted by Krug and Zachmann, which indicated that the injection molded 316L stainless steel powders sintered under nitrogen (N_2_) atmosphere possessed higher tensile strength but lower ductility than those sintered under argon (Ar) and hydrogen (H_2_) atmospheres [98]. Exposure to atmospheric air during the sintering process may lead to contamination to scaffolds and deteriorate their mechanical properties. With porosities larger than 79% and elastic moduli in a range of bone tissue (0.1–30 GPa), titanium scaffolds processed by means of single-step sintering in air possessed a lower compressive strength of (6–9 MPa) [99] than the scaffolds with a porosity of about 70% sintered in vacuum (50–100 MPa) [72] and with flushing high-purity argon gas (15 MPa) [41]. As a consequence, the load bearing capability of titanium scaffolds sintered in air would be quite limited. The importance of furnace construction on the sintering results has also been reported. During sintering, furnace may interact with the sintering atmosphere and consequently influence the sintering results. Krug and Zachmann revealed that by using a molybdenum furnace the carbon content in sintered 316L stainless steel powder could be reduced more than by using a graphite furnace, when a hydrogen atmosphere was used during sintering [98]. In addition, a retort is devised in the furnace design to let it act as a separating chamber to prevent the sintered compacts from contamination during heat treatment or sintering [100].

### 6.3. Determination of Appropriate Sintering Temperatures

Considering the importance of sintering parameters, especially sintering temperature, for the final form and performance of metallic scaffolds, attempts have been made by several researchers to determine appropriate sintering temperatures for scaffold fabrication. Several techniques have been used for the evaluation of optimum sintering temperatures for scaffold fabrication with the space holder method, *i.e.*, (i) compression testing; (ii) observation of inter-particle neck growth and (iii) microstructural analysis. In compression testing, the peak compressive stresses of scaffolds sintered at various temperatures may be used as the basis for evaluation. Hao and co-workers [86] showed that an optimum sintering temperature for magnesium foams was found within a range of 610–630 °C. Magnesium foams with relatively high peak compressive stresses could be made, if sintering took place in this temperature range. However, magnesium foams exhibited low compressive strength due to poor inter-particle bonding, resulting from sintering at temperatures below this critical value. On the other hand, sintering at higher temperatures could distort resulting pore sizes and pore shape due to partial melting. Esen and Bor [42] introduced a technique by observing the neck growth in sintered scaffolds to determine an optimum sintering temperature. In this study, the average neck size or bond diameter formed during sintering (*X*), relative to the average diameter of neighboring particles (*D*) was determined and the results were reproducible. The determination of an optimum sintering temperature based on microstructural observation was introduced by Seyedraoufi and Mirdamadi [63]. In their study, Mg-Zn scaffolds were sintered at temperatures of 500, 550, 565 and 580 °C. Optical microscopic analysis was then performed to examine grain sizes. With this method, it was found that an optimum sintering temperature for Mg-Zn scaffolds was 550 °C. Higher sintering temperatures resulted in grain growth, which would lead to the degradation of scaffolds in mechanical properties [63].

## 7. Concluding Remarks

The space holder method has become a promising one for the fabrication of metallic biomedical scaffolds, owing to its ability to produce a wide range of porosity levels and controllable pore geometry in scaffolds. In principle, four processing steps are involved in this method, *i.e.*, (i) mixing of metal matrix powder and space-holding particles; (ii) compaction of granular materials resulting from the mixing process; (iii) removal of space-holding particles and (iv) sintering of scaffold preform. Prior to mixing, powders must be appropriately selected and prepared. Geometrical and dimensional characteristics, mechanical properties and biocompatibility of powders used in scaffold fabrication are among the critically important parameters of powdered materials that must be considered in scaffold fabrication. During the mixing process, the homogeneity of space-holding particles in the mixture containing metal matrix powder determines the pore distribution and mechanical properties of the resultant scaffolds. Compaction is conducted to achieve an appropriate green strength and improve the sinterability of scaffold preforms. At this step, compacting pressure must be optimized in order to reach a balance between the green strength achieved in scaffold preform and the shape of compacted space-holding particles. Several compaction techniques have been used for scaffold fabrication with the space holder method, such as uniaxial die pressing, isostatic pressing and injection molding. Macro-pores in scaffolds are formed after the removal of space-holding particles, either through heat treatment or leaching in liquid. As the space holder removal process determines the porous structure, green strength and purity of scaffolds, it must be handled carefully. Sintering determines the final form as well as the final porous structure and mechanical properties of scaffolds. With the space holder method, two types of pores are formed in scaffolds, *i.e.*, (i) macro-pores that are formed from the space occupied by space-holding particles and (ii) micro-pores that result from the voids at the interstices of matrix powder particles in the scaffold framework. Sintering temperature and time are among the important technical parameters. Contamination and uncontrolled shrinkage remain the challenges in the sintering process for the fabrication of metallic biomedical scaffolds. Considering all these fabrication parameters, a metallic scaffold with porosity at a level of about 55% is probably the best to be fabricated with the space holder method.

## Figures and Tables

**Table 1.** Bone tissue engineering scaffolds developed from powdered alloys with the space holder method.

Alloyed powder	Method of alloying *	References
Ti-6Al-4V	PA	[42,46]
NiTi	PA, BE	[34,36,55]
Ti-5Mn	BE	[58]
Ti-7.5Mo	BE	[59,60]
Ti-6Ta-4Sn	BE	[57]
Ti-16Sn-4Nb	BE	[65]
316L stainless steel	PA	[66]
AZ91	PA	[62]
Mg-Zn	BE	[63]

* PA = pre-alloying technique; BE = blended elemental technique.

**Table 2.** Space holding particles and considerations in selection for metallic biomedical scaffolds.

Space holder material	Reasons of selection	References
Ammonium hydrogen carbonate	Low decomposition temperature	[33,34,75]
Carbamide	Highly soluble in water	[52,76]
Saccharose	Soluble in water, biocompatible	[39]
Sodium chloride	Soluble in water, biocompatible	[35,36]
Magnesium	Biocompatible, good mechanical properties	[41,42,67]
Steel	Good mechanical properties	[43]

**Table 3.** Mixing process used in the fabrication of metallic biomedical scaffolds with the space holder method. Polyvinyl-alcohol (PVA); polyethylene-glycol (PEG); polymethyl metacrylate (PMMA).

Metal matrix powder	Space holder	Mixer type	Binder	Duration of mixing	References
Titanium	Ammonium hydrogen carbonate	Manual	not defined	3–4 min	[33]
Titanium	Ammonium hydrogen carbonate	V-blender	not defined	8 h	[34]
Titanium	Carbamide	V-blender	PEG	1 h	[28]
Titanium	Carbamide	not defined	Water	1 min	[29]
Titanium, NiTi alloy	Magnesium	not defined	PVA	30 min	[41,42,67,69]
Titanium	Sodium chloride	Turbula mixer	not defined	≥40 min	[35]
Ti-6Al-4V alloy	Carbamide	rolling mixer	Ethanol	1 h	[46,87]
316L stainless steel	Carbamide	Sigma blade mixer	PMMA	30 min	[66]
Stainless steel	Carbamide	Turbula mixer	Paraffin wax	1 h	[52]
Magnesium	Carbamide	Manual	Paraffin powder and ethanol	not defined	[86]

**Table 4.** Experimental determination of optimum compacting pressures for the fabrication of metallic scaffolds with the space holder method.

Metal matrix powder	Space holder	Compacting pressure (MPa)	Method of evaluation	References
Stainless steel	Carbamide	100	Visual inspection	[66]
Titanium	Carbamide	<500	Visual inspection	[29]
Titanium	Corn starch dextrin	400	Visual inspection	[38]
Ti-6Al-4V alloy	Carbamide	450	Visual inspection	[30]
Titanium	Carbamide	250	Microhardness distribution	[31]
Titanium	Carbamide	200	Shrinkage and compressive yield strength of the scaffold	[90]

**Table 5.** Decomposition and removal temperatures of space holders.

Space holder material	Decomposition temperature (°C)	Removal temperature (°C)	References
Ammonium hydrogen carbonate	60	150–175	[33,75]
Carbamide	133	>600	[75]
Tapioca starch	–	450	[37]

**Table 6.** Space holders and solvents for water leaching.

Space holder material	Solvent	References
Carbamide	water, NaOH	[29,52,66,86]
Sodium chloride	water	[35,36,55]
Corn starch dextrin	water	[38]
Saccharose	water	[39]
Magnesium	HCl	[72]
Stainless steel	acetic acid	[43]
